# Incidence rate and clinical impacts of arrhythmia following COVID-19: a systematic review and meta-analysis of 17,435 patients

**DOI:** 10.1186/s13054-020-03368-6

**Published:** 2020-12-10

**Authors:** Shu-Chen Liao, Shih-Chieh Shao, Chi-Wen Cheng, Yung-Chang Chen, Ming-Jui Hung

**Affiliations:** 1 Department of Emergency Medicine, Keelung Chang Gung Memorial Hospital, Keelung, Taiwan; 2 College of Medicine, Chang Gung University, Taoyuan, Taiwan; 3 School of Pharmacy, Institute of Clinical Pharmacy and Pharmaceutical Sciences, College of Medicine, National Cheng Kung University, Tainan, Taiwan; 4 Department of Pharmacy, Keelung Chang Gung Memorial Hospital, Keelung, Taiwan; 5 Section of Cardiology, Department of Internal Medicine, Keelung Chang Gung Memorial Hospital, No. 222, Maijin Rd., Anle Dist., Keelung, Taiwan; 6 Section of Nephrology, Department of Internal Medicine, Linkou Chang Gung Memorial Hospital, Taoyuan, Taiwan; 7 Community Medicine Research Center, Keelung Chang Gung Memorial Hospital, Keelung, Taiwan

Arrhythmia is a potential cardiovascular complication of Coronavirus Disease 2019 (COVID-19) [1]. In one case series of patients hospitalized with COVID-19, 16.7% developed unspecified arrhythmia [2], while another case series indicated sustained ventricular tachycardia or ventricular fibrillation among 5.9% of patients hospitalized with COVID-19 [3]. However, incidence rates of arrhythmia and mortality rates after incident arrhythmia in COVID-19 patients have not been systematically established.

We searched for relevant studies cited in PubMed or Embase up to September 15, 2020, using the terms “COVID-19”, “arrhythmia”, “incidence”, “mortality,” and “prognosis” with suitable MeSH terms. All studies were selected and reviewed by two reviewers (SCL and SCS). The final list of included studies and data extractions were derived through extensive discussion with agreement from both authors. Statistical analyses were performed using MedCalc (Windows) version 15.0 (MedCalc Software, Ostend, Belgium). Outcomes were reported as proportions with 95% confidence interval (CI), based on the random effects model. The heterogeneity among studies was detected by the Cochran *Q* test with *p* value and the *I*^2^ statistic.

Of 645 potential studies screened, we excluded 143 duplicate studies, 66 irrelevant studies, 12 conference abstracts, 241 other types of publications (e.g., pre-prints, protocols, opinions, recommendations, editorials, commentaries, retractions and reviews), 114 studies without incidence or mortality data, and 13 non-English studies. We included 56 studies from 11 countries comprising 17,435 patients with COVID-19. Study characteristics for included articles are listed in Table 1. Notably, most studies only included hospitalized patients with COVID-19 (96.4%). The overall incidence of arrhythmia in COVID-19 patients was 16.8% (95% CI: 12.8–21.2%; *I*^2^: 98.0%, *p* < 0.001) (Fig. 1a). The incidence of different types of arrhythmia in patients with COVID-19 was as follows: 12.0% (22 studies, 95% CI: 8.6–15.9%) for non-classified arrhythmia, 8.2% (14 studies, 95% CI: 5.5–11.3%) for atrial fibrillation/atrial flutter/atrial tachycardia, 10.8% (26 studies, 95% CI: 6.6–15.9%) for conduction disorders, 8.6% (5 studies, 95% CI: 4.5–13.9%) for premature contraction and 3.3% (16 studies, 95% CI: 1.9–4.9%) for ventricular fibrillation/ventricular tachycardia. We found the mortality was 20.3% (95% CI: 12.9–29.0%; *I*^2^: 72.8%, *p* < 0.001) in COVID-19 patients who developed arrhythmia (Fig. 1b).

**Table 1:** Study characteristics

Author/Year	Country	Study design	Setting	Male	Age	HF	CAD	Medication
Du Y/2020	Wuhan/China	Retrospective (2 centers)	Inpatient	72.9	65.8^a^	NA	11.8	Anti-influenza drugs: 77.6; Lopinavir-Ritonavir: 12.9
Wang D/2020	Wuhan/China	Retrospective (1 center)	Inpatient	54.3	56.0	NA	NA	Anti-influenza drugs: 89.9; Azithromycin: 18.1
Guo T/2020	Wuhan/China	Retrospective (1 center)	Inpatient	48.7	58.5 ^a^	NA	11.2	Anti-influenza drugs: 88.8
Rosenberg ES/2020	New York/USA	Retrospective (multicenter)	Inpatient	59.7	63.0	6.7	12.0	Hydroxychloroquine: 18.8; Azithromycin: 14.7; Hydroxychloroquine + Azithromycin: 51.1
Lei S/2020	Wuhan/China	Retrospective (3 centers)	Inpatient	41.2	55.0	NA	NA	Lopinavir-Ritonavir: 100
Saleh M/2020	New York/USA	Prospective (3 centers)	Inpatient	57.2	58.5 ^a^	7.5	11.4	Hydroxychloroquine/Chloroquine: 40.8; (Hydroxychloroquine/Chloroquine) + Azithromycin: 59.2
Chang D/2020	New York/USA	Prospective (1 center)	Inpatient	59.5	60.2 ^a^	0.9	5.1	Hydroxychloroquine: 56.4; Hydroxychloroquine + Azithromycin: 43.6
Bhatla A/2020	Philadelphia/USA	Retrospective (1 center)	Inpatient	45.0	50.0 ^a^	13.0	11.0	Hydroxychloroquine: 24.6; Remdesivir: 8.1
Chorin E/2020	New York/USA	Retrospective (2 centers)	Inpatient	75.0	64.0 ^a^	3.0	12.0	Hydroxychloroquine: 100.0; Azithromycin: 100.0
Sabatino J/2020	Catanzaro/Italy	Cross-sectional (multicenter)	Inpatient	52.6	34.7 ^a^	NA	NA	NA
Mani VR/2020	New York/USA	Retrospective (1 center)	Inpatient	60.3	64.7 ^a^	NA	20.1	Hydroxychloroquine: 21.7; Azithromycin: 12.5; Hydroxychloroquine + Azithromycin: 48.9
Si D/2020	Wuhan/China	Retrospective (1 center)	Inpatient (died)	63.6	64.0	NA	17.4	Azithromycin: 0.8; Anti-influenza drugs: 71.9; Lopinavir- Ritonavir: 7.4; Remdesivir: 0.0
			Inpatient (alive)	32.7	61.5	NA	8.4	Azithromycin: 2.6; Anti-influenza drugs: 83.7; Lopinavir- Ritonavir: 14.3; Remdesivir: 2.0
Angeli F/2020	Varese/Italy	Retrospective (1 center)	Inpatient	72.0	64 ^a^.0	6.0	10.0	Hydroxychloroquine: 82.0; Macrolides: 56.0; Lopinavir- Ritonavir: 54.0
Samuel S/2020	New York/USA	Retrospective (1 center)	Inpatient	57.5	12.6 ^a^	NA	NA	Hydroxychloroquine: 44.0; Hydroxychloroquine + Azithromycin: 25.0; Remdesivir: 5.6; Tocilizumab: 5.6
Ramireddy A/2020	Los Angeles/USA	Retrospective (1 center)	Inpatient	61.0	62.3 ^a^	20.0	NA	Hydroxychloroquine: 10.2; Azithromycin: 27.6; Hydroxychloroquine + Azithromycin: 62.2
Sala S/2020	Milan/Italy	Cross-sectional (multicenter)	Inpatient	66.0	65.0	NA	7.0	Hydroxychloroquine: 100.0; Azithromycin: 100.0
Cao B/2020	Beijing/China	Randomized controlled trial (1 center)	Inpatient	60.3	58.0	NA	NA	Lopinavir-Ritonavir: 49.7
Goyal P/2020	New York/USA	Retrospective (2 centers)	Inpatient	60.6	62.2	NA	13.7	NA
Cao J/2020	Wuhan/China	Retrospective (1 center)	Inpatient	52.0	54.0	NA	NA	Antiviral drugs: 98.0
Zhang G/2020	Wuhan/China	Retrospective (1 center)	Inpatient	48.9	55.0	NA	NA	Antiviral drugs: 88.7
Jun Wu/2020	Wuhan/China	Retrospective (1 center)	Inpatient	54.5	62.0	NA	NA	Antiviral drugs: 97.0
Fernández-Ruiz M/2020	Madrid/Spain	Retrospective (1 center)	Inpatient/outpatient	77.8	71.0	NA	22.2	Lopinavir-Ritonavir + Hydroxychloroquine: 44.4; Lopinavir-Ritonavir: 5.6; Hydroxychloroquine: 27.8
McCullough SA/2020	New York/USA	Retrospective (1 center)	Inpatient	63.2	64.0	7.3	14.4	NA
Lim JH/2020	Daegu/Korea	Retrospective (2 centers)	Inpatient	66.7	75.0	6.7	NA	Hydroxychloroquine: 83.3; Lopinavir-Ritonavir: 96.7
Maraj I/2020	Connecticut/USA	Retrospective (1 center)	Inpatient	56.0	62.7 ^a^	NA	14.0	Hydroxychloroquine: 100.0; Azithromycin: 100.0
Shao F/2020	Wuhan/China	Retrospective (1 center)	Inpatient	66.2	69.0 ^a^	NA	11.0	NA
Lagier JC/2020	Marseille/France	Retrospective (multicenter)	Inpatient/outpatient	45.6	45.0 ^a^	NA	NA	Hydroxychloroquine: 2.7; Azithromycin: 3.7; Hydroxychloroquine + Azithromycin: 89.3
Jung HY/2020	Daegu/Korea	Retrospective (multicenter)	Inpatient	42.9	63.5 ^a^	NA	NA	Lopinavir-Ritonavir: 100.0; Hydroxychloroquine: 50.0
Dubernet A/2020	Réunion Island/France	Retrospective (1 center)	Inpatient	69.4	66.0	NA	NA	Hydroxychloroquine + Azithromycin: 63.9; Lopinavir- Ritonavir: 5.6
Voisin O/2020	Paris/France	Retrospective (1 center)	Inpatient	55.2	68.0	NA	NA	Hydroxychloroquine + Azithromycin: 100.0
Mazzanti A/ 2020	Pavia/Italy	Prospective (multicenter)	Inpatient	63.0	69.0	NA	NA	Hydroxychloroquin:100.0; Hydroxychloroquine + Azithromycin: 26.0; Hydroxychloroquin + Lopinavir- Ritonavir: 35.0; Hydroxychloroquine + Azithromycin + Lopinavir-Ritonavir: 6.0
Gupta MD/2020	New Delhi/India	Case series (1 center)	Inpatient	57.1	56.0	14.3	28.6	NA
Chinitz JS/2020	New York/USA	Retrospective (1 center)	Inpatient	42.9	64.0 ^a^	NA	NA	NA
Ferguson J/2020	California/USA	Retrospective (2 centers)	Inpatient	52.8	60.4	6.9	9.7	Hydroxychloroquine: 22.2; Azithromycin: 45.8; Remdesivir: 44.4; Tocilizumab: 5.6
Argenziano MG/2020	New York/USA	Retrospective (1 center)	Inpatient	60.1	63.0	10.7	13.5	Hydroxychloroquine: 63.9; Azithromycin: 47.6; Remdesivir: 2.1; Tocilizumab: 6.0
Khamis F/2020	Muscat/Oman	Prospective (2 centers)	Inpatient	85.0	48.0 ^a^	NA	NA	Hydroxychloroquine/Chloroquine: 97.0; Azithromycin: 71.0; Lopinavir-Ritonavir: 59.0; Tocilizumab: 3.2
Russo V /2020	Naples/Italy	Retrospective (multicenter)	Inpatient	61.1	66.9 ^a^	11.1	15.9	NA
Xu H/2020	Sichuan/China	Retrospective (1 center)	Inpatient	49.0	NA	NA	NA	Antiviral drugs: 100.0
Chen L/2020	Guangdong/China	Retrospective (3 centers)	Inpatient	67.0	59.5 ^a^	NA	NA	Antiviral drugs: 96.0
Kelly M/2020	Dublin/Ireland	Retrospective (1 center)	Inpatient	61.9	NA	NA	NA	Hydroxychloroquine + Azithromycin: 61.2
Rivinius R/2020	Heidelberg/Germany	Retrospective (multicenter)	Inpatient	81.0	58.6 ^a^	100.0	NA	Hydroxychloroquine: 14.3; Azithromycin: 19.0
Aversa M/2020	New York/USA	Retrospective (1 center)	Inpatient	50.0	65.0	NA	NA	Hydroxychloroquine: 84.0; Azithromycin: 75.0; Remdesivir: 9.0; Tocilizumab: 19.0
Wang ZH/2020	Wuhan/China	Retrospective (1 center)	Inpatient	64.4	67.4 ^a^	NA	NA	Antiviral drugs: 88.1; Lopinavir-Ritonavir: 10.2
Li J/2020	Wuhan/China	Retrospective (1 center)	Inpatient	47.0	58.0	NA	6.0	Antiviral drugs: 78.4
Rey JR/2020	Madrid/Spain	Retrospective (1 center)	Inpatient	54.8	62.3 ^a^	4.9	6.5	Hydroxychloroquine: 77.4; Azithromycin: 45.6; Lopinavir- Ritonavir: 10.4; Tocilizumab: 7.4
Riker RR/2020	Portland/USA	Retrospective (1 center)	Inpatient	100.0	70.0	0.0	33.3	Hydroxychloroquine: 66.6; Azithromycin: 100.0; Remdesivir: 33.3; Tocilizumab: 33.3
Beyls C/2020	Amiens Cedex/France	Retrospective (1 center)	Inpatient	68.3	NA	NA	NA	Lopinavir-Ritonavir: 100
Sheth V/2020	New York/USA	Retrospective (1 center)	Inpatient	71.0	69.0	NA	NA	Hydroxychloroquine: 84.0; Azithromycin: 90.0; Remdesivir: 3.2
Ferrando C/2020	Barcelona/Spain	Prospective (multicenter)	Inpatient	66.8	64.0	1.4	NA	Hydroxychloroquine: 90.1; Azithromycin: 74.8; Lopinavir- Ritonavir: 65.2; Remdesivir: 2.9; Tocilizumab: 42.5
Farré N/2020	Barcelona/Spain	Retrospective (1 center)	Inpatient	57.1	NA	5.3	NA	Hydroxychloroquine: 2.6; Azithromycin: 1.6; Hydroxychloroquine + Azithromycin: 93.3; Tocilizumab: 16.9
Sridhar AR/2020	Washington/ USA	Retrospective (1 center)	Inpatient	60.0	62.0 ^a^	16.0	13.0	Hydroxychloroquine: 100.0
Sekhavati E/2020	Tehran/Iran	Randomized controlled trial (1 center)	Inpatient	50.0	54.3 ^a^	NA	NA	Azithromycin: 100.0; Lopinavir-Ritonavir: 100.0
Satlin MJ/2020	New York/USA	Retrospective (2 centers)	Inpatient	63.0	62.0	9.0	18.0	Hydroxychloroquine: 100.0; Azithromycin: 18.0; Remdesivir: 7.2
Chen L/2020	Wuhan/China	Retrospective (1 center)	Inpatient	76.2	53.0	NA	6.3	Antiviral drugs: 90.5
Oates CP/2020	New York/USA	Retrospective (1 center)	Inpatient	55.0	69.0	NA	19.0	Hydroxychloroquine: 87.0; Azithromycin: 60.0; Remdesivir: 4.0; Tocilizumab: 4.0
Enzmann MO/2020	Dakota/USA	Retrospective (3 centers)	Inpatient	56.7	56.0	10.7	NA	Hydroxychloroquine: 6.0; Hydroxychloroquine + Azithromycin: 44.0; Lopinavir-Ritonavir: 2.0; Tocilizumab: 8.0

*CAD* coronary artery disease, *HF* heart failure, *NA* not reported

^a^In studies not reporting the median, results are represented by the mean

**Fig. 1:**
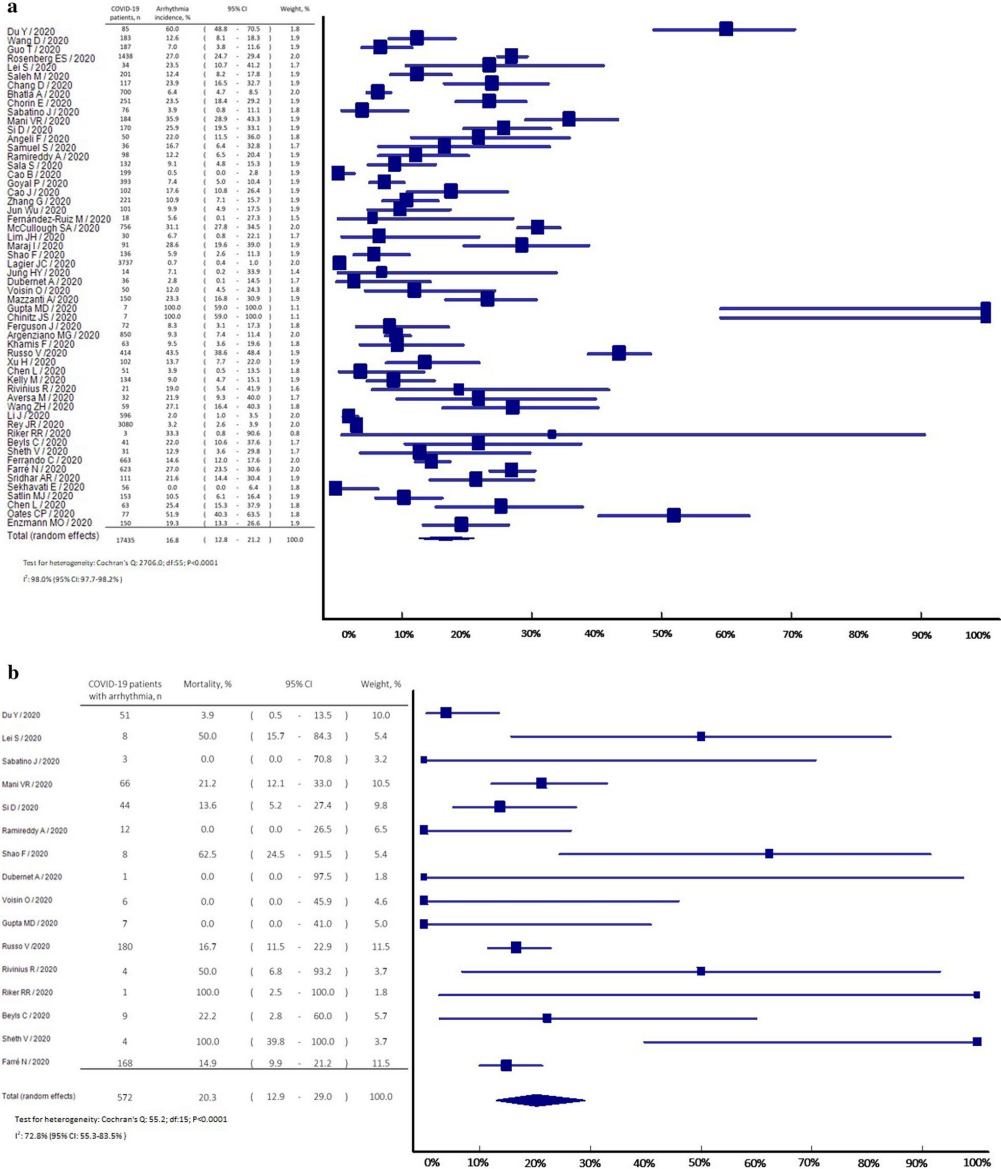
Forest plot of **a** arrhythmia incidence in COVID-19 infections and **b** mortality in COVID-19 patients with incident arrhythmia from included studies

Compared to the incident arrhythmia in patients with community-acquired pneumonia (4.7%, 95% CI: 2.4–8.9) [4], the present study indicates higher incidence of arrhythmia in COVID-19 patients (16.8%) with 2 out of 10 patients dying after developing arrhythmia. The possible mechanisms of arrhythmia may involve cardiac damage from metabolic disarray, hypoxia, neuro-hormonal or inflammatory stress and infection-related myocarditis in the setting of COVID-19 [5]. Notably, higher incidence rates of conduction disorders and premature contraction were found in COVID-19 patients, compared to other types of arrhythmia, in the present study. Our findings increase clinical awareness of arrhythmia in patients hospitalized with COVID-19 for the benefit of first-line healthcare providers.

The major limitation of our study was the inclusion of studies largely from observational data with the potential risk of selection bias. For example, nearly all included studies analyzed data from inpatient settings rather than from the community, likely resulting in overestimation of the true incidence and mortality of arrhythmia among COVID-19 infections. In addition, heterogeneity within and between countries may have caused differences in the estimated incidence and clinical impacts of arrhythmia. Finally, due to the involvement of multiple factors, mortality in COVID-19 patients who developed arrhythmia cannot be entirely attributed to arrhythmia alone. However, the strength of the present study is to summarize the current evidence regarding arrhythmia and COVID-19 infection from various populations worldwide. Since COVID-19 infection probably poses increased risk of arrhythmia, significantly affecting mortality, physicians should consider arrhythmia monitoring with early management in addition to supportive care and respiratory support when treating COVID-19 patients.

## Data Availability

Not applicable.
